# Xylophagia (paper eating): A rare form of pica

**DOI:** 10.1002/ccr3.3157

**Published:** 2020-07-15

**Authors:** Daniel Kurtz, Ibrahim Azar, Hajra Khan, Kirthi Lilley, Indryas Woldie

**Affiliations:** ^1^ Department of Internal Medicine Wayne State University School of Medicine and Detroit Medical Center Detroit MI USA; ^2^ Department of Oncology Karmanos Cancer Center Wayne State University Detroit MI USA; ^3^ Department of Gastroenterology Wayne State University School of Medicine and Detroit Medical Center Detroit MI USA

**Keywords:** Xylophagia, Pica, Iron deficiency anemia

## Abstract

Xylophagia is a form of pica where patients have the unusual craving for ingestion of paper. After treating the underlying cause of pica, in this case treating iron deficiency anemia with iron replacement therapy, these unusual cravings resolve.

## Graphical abstract

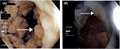

A 32‐year‐old woman with a history of homozygous sickle cell anemia (Hgb SS disease) was started on anticoagulation with apixaban 5 mg BID for superior vena cava thrombosis and pulmonary embolism. Other complications of Hgb SS disease included two CVAs and several admissions for pain crises. She was enrolled in a monthly exchange transfusion program and was on folic acid supplementation. While on anticoagulation for her PE and SVC thrombosis, she developed hematochezia and workup revealed iron deficiency anemia (ferritin 8.1 ng/mL [11‐306.8 ng/mL], hemoglobin 7.0 gm/dL [11.5‐15.1 gm/dL], MCV 77.4 fl [82‐97 fl]). Colonoscopy revealed extensive foreign body, throughout the colon making it difficult to advance the colonoscope beyond the sigmoid colon. Patient was given additional polyethylene glycol 3350 and electrolytes followed by repeat colonoscopy with assistance from large volume of water evacuation, which showed small fragments of toilet paper from the sigmoid colon (Figure 1A arrow) up to the terminal ileum better visualized after removal from the colon (Figure 1B arrow). No active source of bleeding was identified. Upon further questioning, patient reported pica and craving for toilet paper and endorsed eating up to two rolls of toilet paper daily. She received three doses of intravenous iron replacement therapy with normalization of her ferritin (89.3 ng/mL [11‐306.8 ng/mL]). Upon follow‐up of 8 weeks after iron replacement therapy, her cravings had completely resolved and she was no longer eating toilet paper. Xylophagia is a form of pica where patients have the unusual craving for ingestion of paper.^1^, ^2^

**FIGURE 1:**
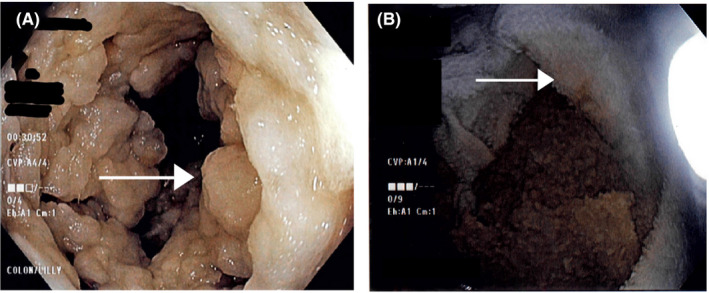
A, Sigmoid colon filled with fragments of toilet paper (arrow). B, Toilet paper after removal from colon (arrow)

## CONFLICT OF INTEREST

None declared.

## AUTHOR CONTRIBUTION

DK and IA: wrote the manuscript. HK and KL: provided high‐quality images. All authors, including IW: reviewed the final manuscript.
